# *ERBB2*‐amplified lobular breast carcinoma exhibits concomitant *CDK12* co‐amplification associated with poor prognostic features

**DOI:** 10.1002/2056-4538.12362

**Published:** 2024-01-21

**Authors:** Miriam Forster‐Sack, Martin Zoche, Bernhard Pestalozzi, Isabell Witzel, Esther Irene Schwarz, Joel Julien Herzig, Hisham Fansa, Christoph Tausch, Jeff Ross, Holger Moch, Zsuzsanna Varga

**Affiliations:** ^1^ Department of Pathology and Molecular Pathology University Hospital Zurich Zürich Switzerland; ^2^ Department of Oncology University Hospital Zurich Zürich Switzerland; ^3^ Comprehensive Cancer Center, Breast Center University Hospital Zurich Zürich Switzerland; ^4^ Department of Gynecology University Hospital Zurich, University of Zurich Zürich Switzerland; ^5^ Department of Pulmonology University Hospital Zurich Zürich Switzerland; ^6^ Breast Center Hospital Zollikerberg Zollikerberg Switzerland; ^7^ Breast Center Seefeld Zürich Switzerland; ^8^ Department of Pathology, Urology and Medicine (Oncology) Upstate Medical University Syracuse NY USA; ^9^ Foundation Medicine, Inc. Cambridge MA USA

**Keywords:** lobular carcinoma, *ERBB2*/*CDK12* coamplification, lobular *ERBB2* mutations, poor prognosis

## Abstract

Most invasive lobular breast carcinomas (ILBCs) are luminal‐type carcinomas with an HER2‐negative phenotype (*ERBB2 or HER2* un‐amplified) and *CDH1* mutations. Rare variants include *ERBB2*‐amplified subtypes associated with an unfavorable prognosis and less response to anti‐HER2 targeted therapies. We analyzed the clinicopathological and molecular features of *ERBB2*‐amplified ILBC and compared these characteristics with *ERBB2*‐unamplified ILBC. A total of 253 patients with ILBC were analyzed. Paraffin‐embedded formalin‐fixed tumor samples from 250 of these patients were added to a tissue microarray. Protein expression of prognostic, stem cell and breast‐specific markers was tested by immunohistochemistry (IHC). Hybrid capture‐based comprehensive genomic profiling (CGP) was performed for 10 ILBCs that were either fluorescent *in situ* hybridization (FISH) or IHC positive for *HER2* amplification/overexpression and 10 ILBCs that were either FISH or IHC negative. Results were compared with a CGP database of 44,293 invasive breast carcinomas. The CGP definition of *ERBB2* amplification was five copies or greater. A total of 17 of 255 ILBC (5%) were *ERBB2* amplified. *ERBB2*‐amplified ILBC had higher tumor stage (*p* < 0.0001), more frequent positive nodal status (*p* = 0.00022), more distant metastases (*p* = 0.012), and higher histological grade (*p* < 0.0001), and were more often hormone receptor negative (*p* < 0.001) and more often SOX10 positive (*p* = 0.005). *ERBB2* short variant sequence mutations were more often detected in *ERBB2*‐unamplified tumors (6/10, *p* = 0.027), whereas *CDH1* mutations/copy loss were frequently present in both subgroups (9/10 and 7/10, respectively). Amplification of pathogenic genes were more common in HER2‐positive ILBC (*p* = 0.0009). *CDK12* gene amplification (≥6 copies) was detected in 7 of 10 *ERBB2*‐amplified ILBC (*p* = 0.018). There were no *CDK12* gene amplifications reported in 44,293 invasive breast carcinomas in the FMI Insights CGP database. *ERBB2*‐amplified ILBC is a distinct molecular subgroup with frequent coamplification of *CDK12*, whereas *ERBB2* sequence mutations occur only in *ERBB2*‐unamplified ILBC. *CDK12*/*ERBB2* co‐amplification may explain the poor prognosis and therapy resistance of *ERBB2*‐amplified ILBC.

## Introduction

Invasive lobular breast carcinomas (ILBCs) represent 10–15% of all invasive breast tumors and, in the majority of cases, express hormone receptors [estrogen receptor (ER) and progesterone receptor (PR)] and lack HER2 protein expression and/or gene amplification [1]. ILBCs are usually clinically poorly defined or diffuse tumors and due to lack of associated calcifications often pose diagnostic imaging difficulties and underestimation of tumor stage [1, 2]. Although luminal‐type lobular carcinomas have potentially favorable features such as an ER/PR positive, HER2‐negative phenotype with low proliferative activity, there is still a controversy whether luminal‐type ILBCs do differ in outcome from stage‐matched invasive ductal carcinomas of no special type (NST) [1, 2, 3]. Furthermore, a rare subgroup of HER2‐positive ILBC tends to be of higher histological grade, more often nodal positive and ER/PR negative [4, 5]. These poor prognostic features have been described in association with resistance to anti‐HER2 therapy in the HER2‐positive ILBC subgroup [3, 6]. Molecular and oncological features of luminal type ILBC have been widely studied [3, 7, 8]. However, no data are available on molecular gene alterations and on the immunohistochemical protein expression profile in the HER2‐positive lobular subtype.

In this study, we aimed to analyze a large cohort of ILBC that also included HER2‐positive cases and address the following questions:

1. Is there any clinicopathological difference such as stage, grade, and age between HER2‐negative and HER2‐positive ILBC?
2. Do HER2‐positive ILBCs have a distinct protein expression profile in terms of prognostic, stem‐cell, tumor‐progression, and breast‐specific markers as compared to the HER2‐negative subgroup?
3. What is the molecular background of HER2‐positive ILBC and is the genetic landscape different from HER2‐negative ILBC cases?
4. Is there any correlation between molecular changes and clinicopathological parameters?

For this purpose, we retrospectively analyzed 253 patients with previously diagnosed ILBC, including 15 HER2‐positive cases. Formalin‐fixed and paraffin‐embedded (FFPE) samples were retrieved, a tissue microarray was constructed from 250 of these cases, and selected cases (*n* = 20) underwent DNA‐based FoundationOne®CDx testing to explore the distinct molecular landscape of HER2‐positive ILBC.

## Materials and methods

The Ethical Committee of Canton Zurich permitted the data collection and the further use of archived tissue as this work is part of a larger retrospective breast cancer study (KEK‐2012‐553, BASEC‐2019‐00111).

### Patient cohort

The database of the Department of Pathology and Molecular Pathology at the University Hospital Zurich in Switzerland was searched for patients diagnosed with an invasive or mixed ILBC between 2000 and 2019. Further inclusion criteria, in addition to supporting immunohistochemistry (IHC) (E‐cadherin/catenin p120), were complete information on clinical and/or pathological tumor stage, results of intrinsic parameter assessment done in this institution (such as ER, PR, HER2, and Ki67 index), female gender, available follow‐up data, and available archived tumor material (biopsy or surgical specimen) as well as documented patient's permission to use their data and biological material for further research purposes. We identified 397 patients, 144 of whom were excluded due to incomplete inclusion criteria; 253 patients were included in this study, 250 of them included in a tissue microarray construction.

### Patient characteristics

We identified 253 female patients who were diagnosed with ILBC in pure form or in combination with invasive ductal (NST) carcinoma. We divided the patients into two groups depending on their slide‐based *ERBB2* amplification or HER2 protein expression status: 15 patients were HER2 positive and 238 patients were HER2 negative.

**Figure 1:**
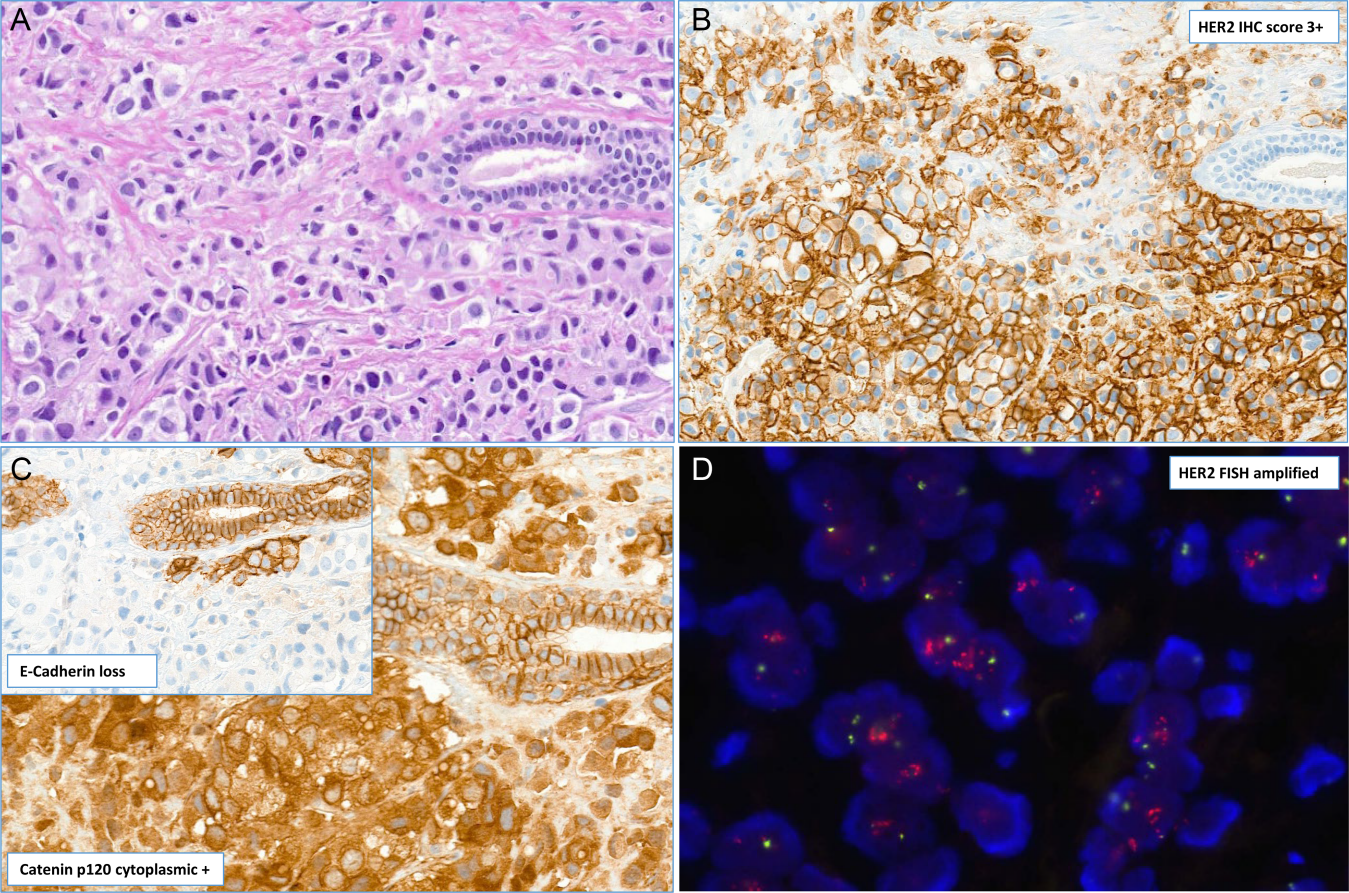
Illustration of morphology, immunophenotype, and HER2 status assessment in an HER2‐positive invasive lobular carcinoma. (A) Dyscohesive spread of invasive carcinoma cells without evidence of gland formation showing wide cytoplasm and moderate to high nuclear pleomorphism (H&E stain). (B) HER2 immunohistochemistry shows strong membrane staining in >10% of the tumor surface corresponding to HER2 Score 3+ (HER2 IHC). (C) Immunohistochemical stains demonstrate cytoplasmic staining for Catenin p120 and loss of E‐Cadherin (Inset) in the tumor cells, while the local ductular structures as internal control are membrane positive in both stains. (D) Fluorescent *in situ* hybridization (FISH) reveals clusters of the HER2 gene (orange/red) and 1–2 copies of the centromeric region of chromosome 17 (green dots) corresponding to a positive gene amplification status.

**Figure 2:**
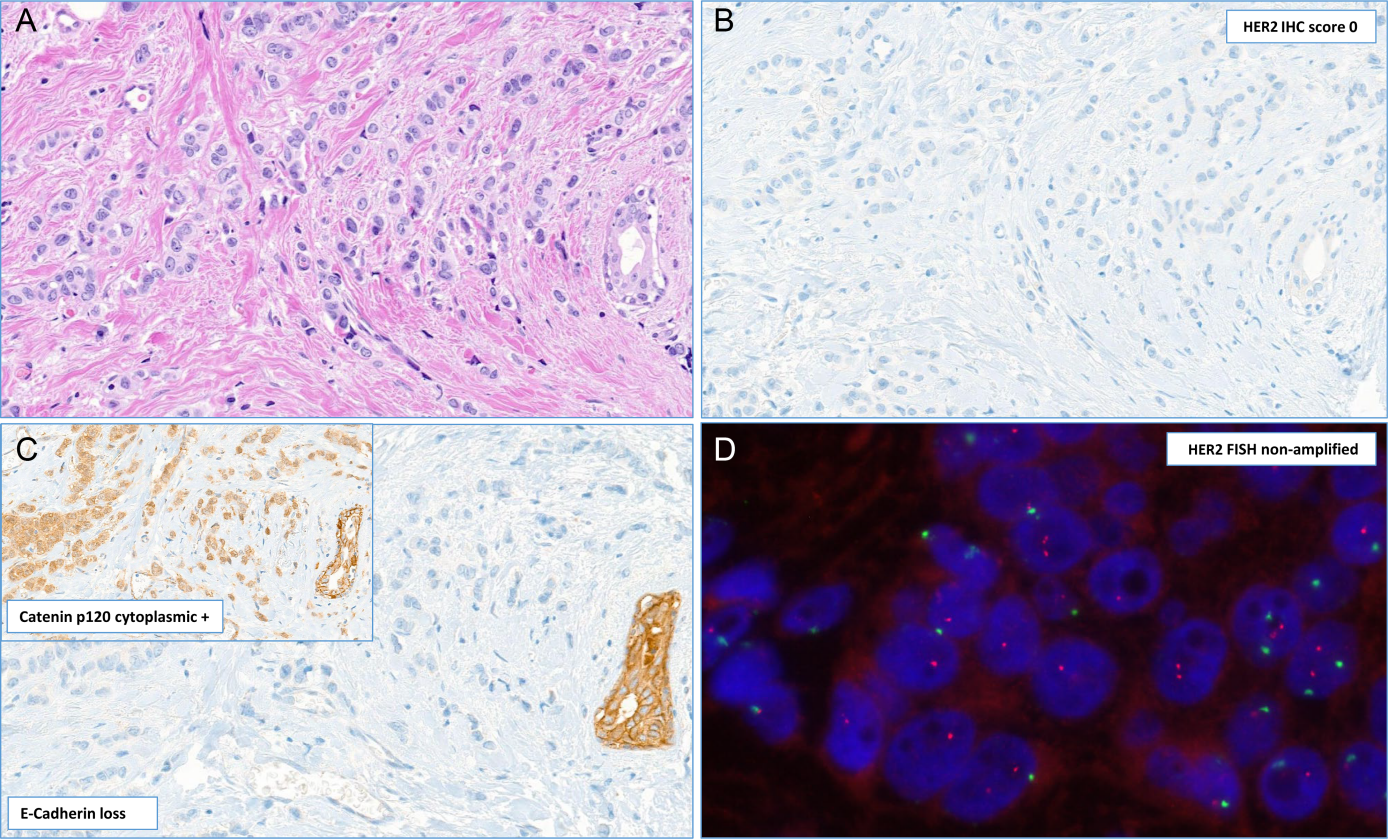
Illustration of morphology, immunophenotype, and HER2 status assessment in an HER2‐negative invasive lobular carcinoma. (A) Dyscohesive spread of invasive carcinoma cells with a periductular targetoid pattern showing scant cytoplasm and moderate pleomorphism (H&E stain). (B) HER2 immunohistochemistry lacking any membranous staining corresponding to HER2 Score 0 (HER2 IHC). (C) Immunohistochemical stains demonstrate complete loss of E‐cadherin membrane staining and strong cytoplasmic staining for Catenin p120 (Inset) in the tumor cells, while the local ductular structures as internal control are membrane positive in both stains. (D) Fluorescent *in situ* hybridization (FISH) reveals diploid tumor cells with two copies of the HER2 gene illustrated with orange/red color and 1–2 copies of the centromeric region of chromosome 17 (green dots) corresponding to a negative gene amplification status.

HER2 status was defined as score 3+ on IHC and/or amplified HER2 status by fluorescent *in situ* hybridization (FISH) as defined by the current American Society of Clinical Oncology (ASCO)/College of American Pathologists (CAP) guidelines [9, 10, 11]. All 15 (100%) of the ‘HER2‐positive’ ILBC were *ERBB2* amplified by FISH testing as all of these cases underwent double testing for HER2 (both IHC and FISH) according to the institute's internal guidelines as described previously [12, 13, 14]. The histological diagnosis of invasive lobular carcinoma was in line with the third, fourth, and fifth editions of the WHO classification of breast tumors consisting of a proliferation of small cells arranged in single files or in solid structures in many cases with a targetoid pattern without any glandular differentiation [1, 15, 16]. In most cases, there was diagnostic confirmation by IHC with loss of E‐cadherin membrane staining (146/253 cases) and/or catenin p120 cytoplasmic staining (239/253) in accordance with the 2019 WHO classification [1, 15, 16]. All cases included in the study underwent a retrospective review, the diagnosis of an invasive lobular carcinoma was reconfirmed, and an appropriate tumor block [available in 12 cases in the HER2 positive subgroup and in 238 cases in the HER2‐negative group for the tissue microarray (TMA) construction] with sufficient tissue for the punches was selected (Figures 1 and 2).

The *ERBB2‐*amplified tumors (*n* = 15) had a mean age at diagnosis of 55 years (range, 30–72 years). Based on the current TNM‐Classification, six tumors were staged as pT1 (40%), two patients (13%) as pT2, five patients (33%) as pT3, and two patients (13%) as pT4 [1]. Five patients were node negative (33%) and 10 presented with axillary lymph node metastases (67%, 7 with pN1, 3 with pN3 status). Information on distant metastases was available in this subgroup in 12 of 15 patients: 4 of 15 patients had distant metastases (osseous, peritoneal, or hepatic) (27%), and 8 of 15 cases did not have documented distant metastases (53%). Seven of 15 patients had local recurrence (47%), and 8 of 15 patients had no documented recurrent disease (53%). Twelve patients had pure invasive lobular carcinoma (three classical and nine pleomorphic or alveolar type) (75%), and three patients (25%) had classical lobular carcinoma in combination with ductal (NST) carcinoma according to the WHO classification [1]. Histological grading was either grade 2 (moderately differentiated, 7 of 15, 46%) or grade 3 (poorly differentiated, 8 of 15, 54%). ER was positive in 10 of 15 patients (67%) and negative in 5 of 15 (33%) patients. PR was positive in 6 of 15 (40%) patients and negative in 9 of 15 patients (40%), using 1% cutoff for positivity for both ER and PR [17]. A total of 5 of 15 ILBCs had lobular carcinoma in situ (LCIS) (pleomorphic type) in the vicinity of the invasive carcinoma cells.

The *ERBB2‐*unamplified group included 238 patients. The mean age at diagnosis was 62 years (range 31–92 years). A total of 65 of 238 patients (27%) were staged as pT1, 93 of 238 (39%) patients were staged as pT2, 41 of 238 (17%) patients were staged as pT3, and 3 of 238 (1%) had pT4 tumors. In 36 cases (15%), pT information was not available. A total of 105 of 238 (44%) patients were nodal negative, 52 of 238 (22%) patients were staged as pN1, 18 patients (8%) were staged as pN2, and 16 (7%) patients were staged as pN3. Information on pN stage was missing in 47 cases (20%). Data on distant metastasis were available in 142 of 238 (60%) patients. A total of 22 of these 142 (15%) patients had distant metastases (osseous, hepatic, visceral, or disseminated), and 120 of 142 patients (85%) had no documented distant metastasis. Information on distant metastases was not available in the remaining 96 patients (40%). Most patients (175 of 238, 74%) had moderately differentiated carcinomas (G2); 12 of 238 (5%) were graded as G1 (well differentiated) and 29 of 238 (12%) patients were graded as G3 (poorly differentiated). In 22 cases (9%), no correct grading was possible due to the small tumor amount on the biopsies. Most patients (186 of 238, 78%) had pure classical type of invasive lobular carcinoma; 46 of 238 (19%) patients had a mixed invasive lobular also of pleomorphic/alveolar type and ductal carcinoma (NST) and 6 of 238 (3%) patients had mixed invasive classical lobular and invasive tubular carcinoma. ER was positive in 222 of 238 patients (92%), negative in 8 of 238 (3%) patients, and not available in 8 patients (3%) using a 1% cutoff for positivity for ER and PR [17]. PR was positive in 190 of 238 (80%) patients, negative in 37 of 238 patients (16%), and not available in 11 patients (5%).

Details of clinicopathological parameters are shown in Table 1.

**Table 1:** Clinical and histological characteristics of the patients (*n* = 253)

Clinicopathological parameter	HER2 positive (*N =* 15)	HER2 negative (*N* = 238)	Total (*N* = 253)	*p* values
Mean age at diagnosis – years	55	62	58.5	ns
Tumor stage, *n* (%)				
pT1	6 (40)	65 (27)	71 (28)	
pT2	2 (13)	93 (39)	95 (37)	<0.0001
pT3	5 (33)	41 (17)	46 (18)	
pT4	2 (13)	3 (1)	5 (2)	
Unknown	0 (0)	36 (15)	36 (15)	
Lymph node stage, *n* (%)				
pN0	5 (33)	105 (44)	110 (43)	
pN1	7 (47)	52 (22)	59 (23)	0.00022
pN2	0 (0)	18 (8)	18 (7)	
pN3	3 (20)	16 (6)	19 (8)	
Unknown	0 (0)	47 (20)	47 (19)	
Distant metastasis, *n* (%)				
M0	8 (53)	120 (51)	127 (50)	0.012
M1	4 (27)	22 (9)	26 (10)	
Unknown	3 (20)	96 (40)	100 (40)	
Grading, *n* (%)				
1	0 (0)	12 (5)	12 (5)	
2	7 (46)	175 (74)	182 (71)	
3	8 (54)	29 (12)	37 (15)	<0.0001
NA	0 (0)	22 (9)	22 (9)	
Histological type, *n* (%)				
Pure invasive lobular	12 (80)	186 (78)	198 (78)	
Mixed invasive lobular and NST	3 (20)	46 (19)	49 (20)	ns
Mixed invasive lobular and tubular	0 (0)	6 (3)	6 (2)	
Hormone receptor status, *n* (%)				
Estrogen receptors				
Positive	10 (67)	222 (92)	232 (92)	
Negative	5 (33)	8 (3)	13 (5)	<0.001
Unknown	0 (0)	8 (3)	8 (3)	
Progesterone receptors				
Positive	6 (40)	190 (80)	196 (78)	
Negative	9 (60)	37 (16)	46 (18)	<0.0001
Unknown	0 (0)	11 (4)	11 (4)	

### Tissue microarray construction

FFPE tissue samples from 250 patients (*n* = 12 HER2 positive and *n* = 238 HER2‐negative carcinomas) were punched based on a hematoxylin–eosin (H&E) stained slide with selection of the invasive area. Areas with the highest tumor density were chosen for the TMA. The punched cores were arranged into the TMA stratified by HER2 status. From each patient, two tissue cores were punched from the paraffin block (either with biopsy tissue or surgical specimen) and added to the TMA.

### Immunohistochemistry

Immunohistochemical reactions were performed at the Department of Pathology and Molecular Pathology, University Hospital Zurich.

The following immunohistochemical stains were analyzed:

Prognostic markers: ER, PR, HER2, and Ki‐67.

Tumor progression markers: p53, PTEN, and PIK3CA.

Stem‐cell specific proteins: SOX2, SOX9, SOX10, CD44, SLUG, TWIST, and mTOR.

Breast‐specific markers: E‐cadherin, catenin p120, CK5/6, p63, GATA3, CK7, NY‐BR‐1, and Mammaglobin. Specifications and laboratory details for the respective stains are listed in details in supplementary material, Table S1.

### Scoring of immunohistochemical stains

All immunostains were scored semiquantitatively as follows:

ER, PR, and Ki‐67: the percentages of positively stained nuclei were reported in 10% steps [negative (<1% cutoff), <10%, >10% in 10% steps] as defined in the ASCO/CAP guidelines [17].

HER2: score 0, 1+, 2+, and 3+ as defined in the ASCO/CAP guidelines [11].

p63: two‐tiered score as negative or nuclear positive.

p53: three tiered as negative (aberrant null pattern), wild type, and overexpression type.

SOX2, SOX9, SOX10, CD44, SLUG, and TWIST: three‐tiered scoring system (negative, ≤50% of the cells positive, >50% of the cells positive), being cytoplasmic and/or nuclear positive.

CK5/6 and catenin p120: two‐tiered scoring system (negative versus positive) being membranous and/or cytoplasmic positive.

PTEN: three‐tiered scoring system (negative, ≤50% of the cells positive, >50% of the cells positive), considering cytoplasmic positivity.

E‐cadherin: two‐tiered scoring system (negative/partial loss versus complete positive), considering membranous positivity.

PIK3CA and mTOR: two‐tiered scoring system (negative versus positive), being membranous and/or nuclear positive.

Brst‐2, NY‐BR‐1, and Mammaglobin: three‐tiered scoring system (negative, ≤50% of the cells positive, >50% of the cells positive), being cytoplasmic and/or nuclear positive.

GATA3: three‐tiered scoring system (negative, ≤50% of the cells positive, >50% of the cells positive), being nuclear positive.

CK7: three‐tiered scoring system (negative, ≤50% of the cells positive, >50% of the cells positive), considering membranous/cytoplasmic positivity.

### Comprehensive genomic profiling

Ten ERBB2‐amplified and 10 ERBB2‐unamplified ILBCs (Zurich cohort) were selected for comprehensive genomic profiling (CGP). HER2‐negative cases were selected as control cases with available existing data in the local FMI (Foundation One Molecular Tumor Profiling) data bank serving as negative biological controls. HER2‐negative ILC samples submitted for FMI testing were not stage matched with HER2‐positive ones.

The results were compared with CGP results of 463,546 samples of all tumor types, including 44,293 breast cancer cases of all tumor types (Foundation Medicine cohort). Approval for this portion of the study, including a waiver of informed consent and a Health Insurance Portability and Accountability Act waiver of authorization, was obtained from the Western Institutional Review Board (protocol no. 20152817). Using the submitted pathology reports and corresponding relevant clinical information, the tissue samples were classified as either obtained from metastatic site biopsies or from sites of unresectable locoregional disease.

The comprehensive genomic profile test (FoundationOne®CDx) for testing of 324 cancer‐related genes for the detection of base exchanges, insertions, deletions, and copy number changes was used. DNA was extracted from FFPE tissue blocks with the Maxwell 16 FFPE Plus LEV DNA Purification Kit (AS1135) according to the manufacturer's recommendations. The tumor cell content was at least 20%. The DNA stock concentration was between 50 and 100 ng/μl for further analysis. The Foundation Medicine cohort was analyzed in a Clinical Laboratory Improvement Amendments‐certified, CAP‐accredited laboratory as previously described. The Zurich cohort was analyzed in the Foundation Medicine laboratory at the Department of Pathology and Molecular Pathology, University Hospital Zurich, as previously described. Both laboratories use identical protocols. All cases included in this study were evaluated by an experienced board‐certified pathologist at the time of specimen arrival in the laboratory and then reviewed by a single pathologist to confirm the diagnosis. Sections were macrodissected to achieve ≥20% estimated percent tumor nuclei cellularity, defined as the number of tumor cells divided by the total number of nucleated cells as assessed by light microscopy. Next, ≥55 ng DNA was extracted from FFPE tumor samples. Samples were assayed by adaptor ligation hybrid capture, performed for all coding exons of 309 cancer‐related genes plus select introns from 34 genes. Sequencing was performed using the Illumina HiSeq instrument to a median exon coverage ≥500 Å~ (Angstrom), and data were analyzed for all classes of genomic alterations. The computational pipeline used to analyze sequence patterns used Bayesian algorithms to identify base substitution mutations, local assembly to identify short insertions and deletions, comparisons with process‐matched normal controls to determine gene amplifications and homozygous deletions and the analysis of chimeric read pairs to identify gene rearrangements and gene fusions. For *ERBB2*, amplification was defined at ≥5 copies. For the other 323 genes, amplification was defined at ≥6 copies. Using 0.8–1.1 Mb of sequenced DNA for each case, the tumor mutational burden (TMB) was determined using the number of somatic base substitution or indel alterations per Mb after filtering to remove germline and pathogenic mutations [18]. Scoring of TMB was defined as follows: low mutations ≤5/Mb, intermediate >5 ≤ 20/Mb, high >20 ≤ 50/Mb, and very high >50/Mb [19]. Microsatellite instability (MSI) was defined as stable versus unstable as defined in the test [19]. Loss of heterozygosity (LOH) was defined as the percentage of gene copy loss. Mutations/amplifications /copy loss/re‐arrangements/truncations were specifically reported for each sample [19].

In this manuscript, the attribution of the definition of ‘targetable’ to any single gene or genomic pathways is based on the current knowledge of available therapeutic compounds already approved in any tumor types. Such a definition relies on an informal consensus among authors, based on the review of existing approved testing and therapy combinations, similar to what has been previously reported by the ASCO and the European Society for Medical Oncology, added to emerging clinical trial data [20, 21].

### Statistics

To test significant differences between HER2‐positive and HER2‐negative invasive lobular carcinomas (both in protein expression via IHC and in the number of detected molecular alterations via the FoundationOne test), the Fisher's exact test and Chi‐square tests were applied, using the online tool Chi‐square calculator – up to 5 × 5, with steps (socscistatistics.com). Kaplan–Meier survival analysis (with Cox regression) was performed using Stata V 15.1 (StataCorp, College Station, TX, USA). Log‐rank tests and Cox regression were used to compare the equality of survival functions (visual overview for creating graphs: Kaplan–Meier survival function | Stata).

Tests in Chi‐square were two‐sided, and *p* values <0.05 in all tests were considered statistically significant.

## Results

### Immunohistochemistry

We included a total of 250 patients in the TMA; however, due to partial or complete loss of some tissue cores in the broad panel of IHC, we could only evaluate the material and data of at least 240 patients (at least 11 in the HER2‐positive group and 229 in HER2‐negative group) for each stain.

In summary, IHC showed that the expression of prognostic, tumor progression, stem‐cell, and breast‐specific markers does not show major differences between *ERBB2*‐amplified and *ERBB2*‐unamplified invasive lobular carcinomas. The only significant differences were detected in the expression of ER (*p* < 0.001) and PR (*p* = 0.013), both of which were more frequently negative in the HER2‐positive subgroup. SOX10 showed a reciprocal significant correlation (*p* = 0.0005) with more frequent positivity in the *ERBB2*‐amplified group. There was pleomorphic LCIS in the vicinity of the invasive component in 5 of 12 HER2‐positive ILCs; all these five LCIS areas were also HER2 positive on IHC (score 3+). Despite discrepant case volumes (low number of HER2‐positive cases in contrast to high number of HER2‐negative ones), Chi‐square test reliably differentiated both groups.

**Figure 3:**
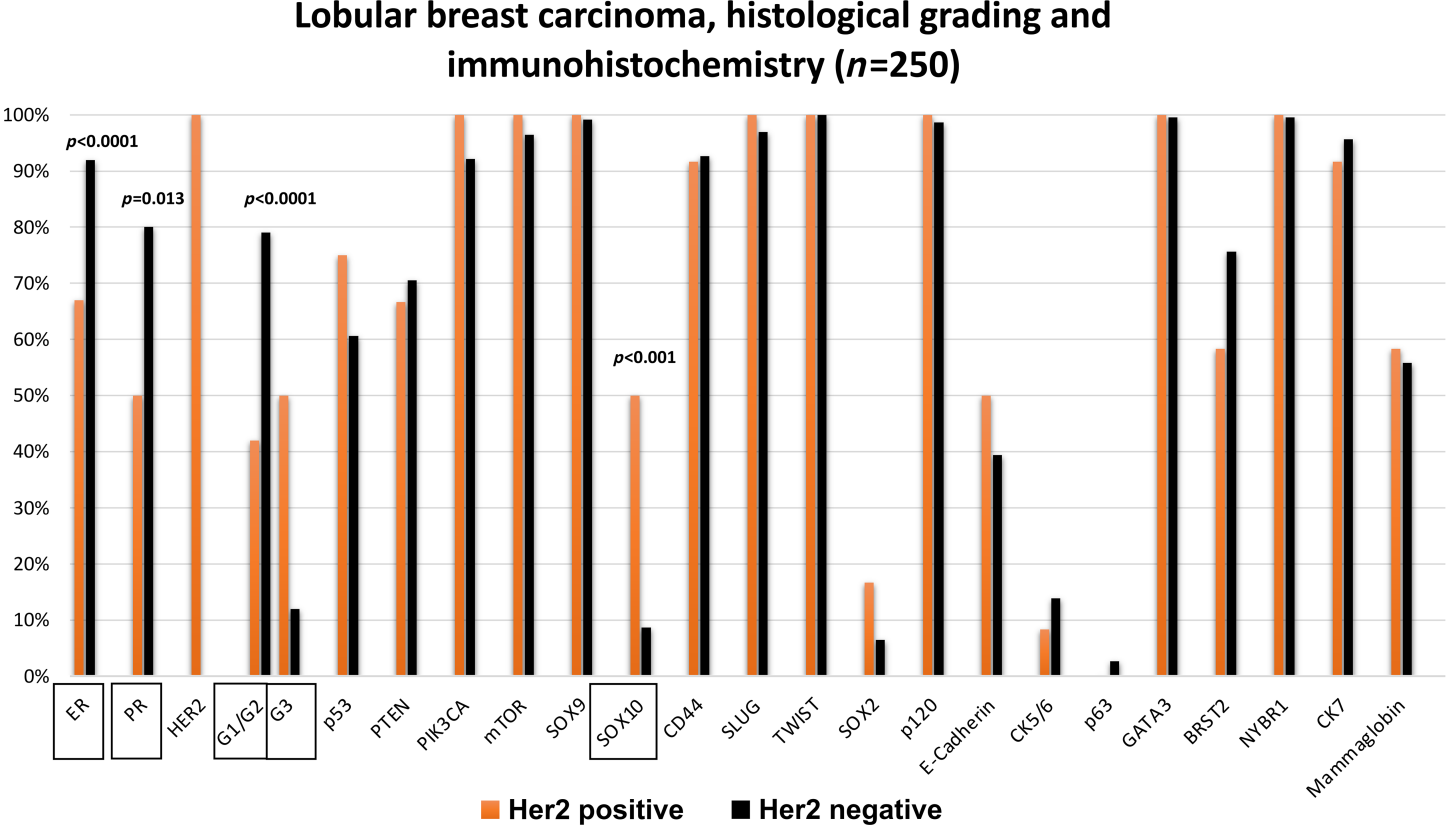
Graphical illustration of the results of the immunohistochemical stains in HER2‐positive (*n* = 12) and HER2‐negative (*n* = 238) invasive lobular carcinomas. The columns show the positively stained samples a percentages. Significant differences were detected in the expression of hormone receptors (*p* < 0.001 resp. *p* = 0.013) and SOX10 (*p* < 0.001). There was also a significant difference in histological grade (*p* < 0.0001).

The results of the immunohistochemical tests are described in detail in Table 2 and graphically illustrated in Figure 3.

**Table 2:** Results of immunohistochemical stains and histological grading on the tissue microarray (*n* = 250 cases)

Clinicopathological parameter	HER2 positive (*N* = 12)	HER2 negative (*N* = 238)	Total	*p* value
ER				
Positive	8	222	242	**<0.0001**
Negative	4	8		
PR				
Positive	6	190	238	**0.013**
Negative	5	37		
HER2				
Positive	12	0	250	
Negative	0	238		
Grading				
G1/G2	5	187	210	**<0.001**
G3	6	12		
p53				
Positive	9	140	243	0.99
Negative	3	91		
PTEN				
Positive	8	163	243	0.77
Negative	4	68		
PIK3CA				
Positive	12	213	243	0.98
Negative	0	18		
mTOR				
Positive	12	221	241	0.60
Negative	0	8		
SOX9				
Positive	12	229	243	0.07
Negative	0	2		
SOX10				
Positive	6	20	243	**0.0005**
Negative	6	211		
CD44				
Positive	11	214	243	0.9
Negative	1	17		
SLUG				
Positive	12	222	241	0.43
Negative	0	7		
TWIST				
Positive	12	231	243	1
Negative	0	0		
SOX2				
Positive	2	15	243	0.17
Negative	10	216		
p120				
Cytoplasmic stain	12	227	242	1
Negative	0	3		
E‐Cadherin				
Partial loss of membranous stain	6	91	243	0.46
Negative	6	140		
CK5/6				
Positive	1	32	243	0.50
Negative	11	199		
p63				
Positive	0	6	240	0.31
Negative	11	223		
GATA3				
Positive	12	229	242	1
Negative	0	1		
BRST2				
Positive	7	174	242	0.18
Negative	5	56		
NYBR1				
Positive	12	229	242	1
Negative	0	1		
CK7				
Positive	11	221	243	0.51
Negative	1	10		
Mammaglobin				
Positive	7	129	243	0.86
Negative	5	102		

Samples with missing/not interpretable spots (not available) on the tissue microarrays are not included in this table; therefore, the absolute number per study group can vary from the cohort number.

### Molecular findings

Altogether 20 FFPE samples were subjected to FoundationOne®CDx testing: 10 patients with *ERBB2*‐amplified lobular carcinoma and 10 patients with *ERBB2*‐unamplified lobular carcinoma.

#### Frequency of pathogenic changes

**Figure 4:**
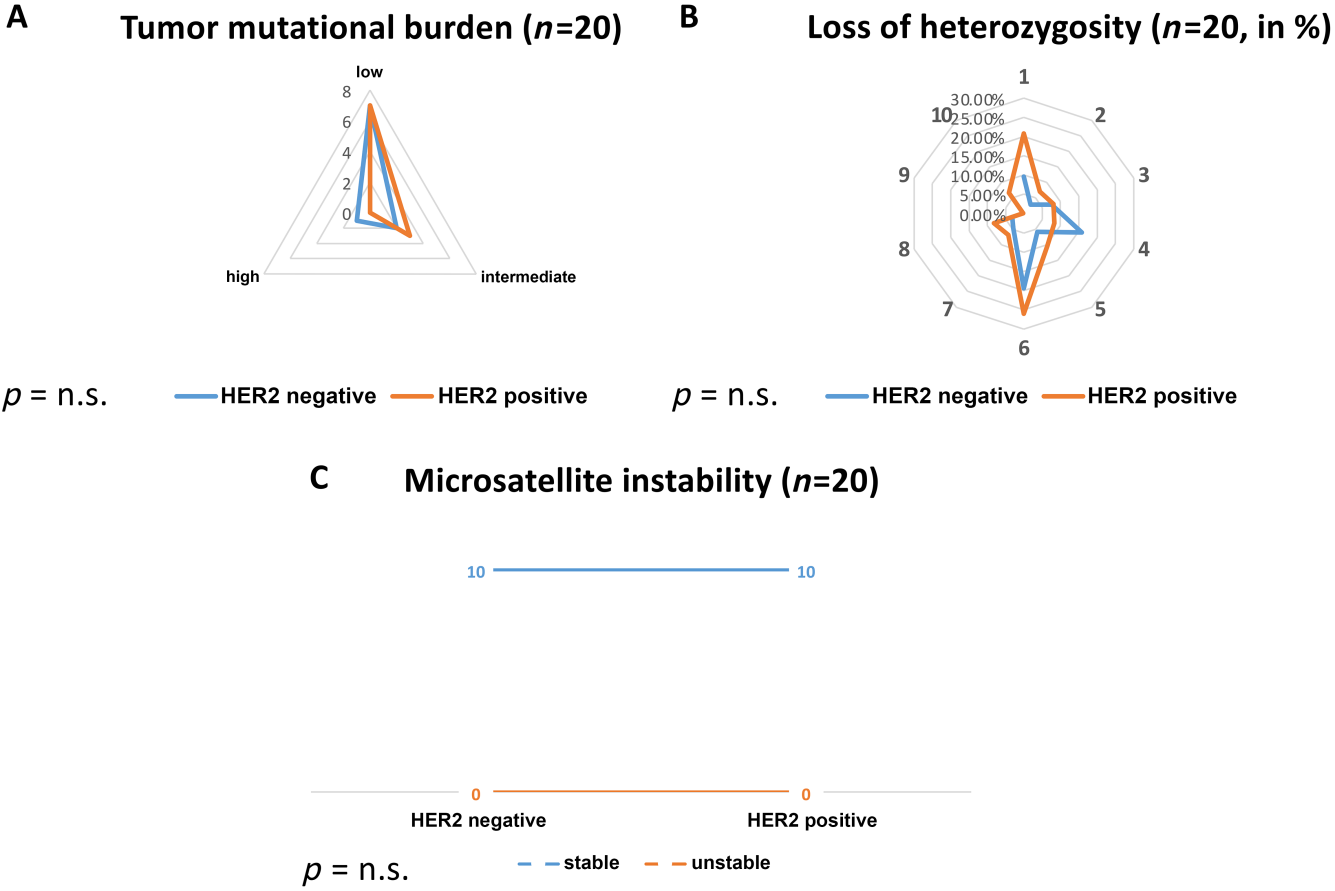
Graphical demonstration of the next‐generation sequencing detection rates of (A) tumor mutational burden, (B) loss of heterozygosity, and (C) microsatellite instability as a comparison between HER2‐positive and HER2‐negative breast carcinomas. The raw data for the graphics are described in detail in Table 3. There was no statistical difference in these parameters between the two groups.

There were on average 101 pathogenic changes in the *ERBB2‐*unamplified subgroup and 165 pathogenic changes (including mutations, gene amplifications, gene copy loss, and gene rearrangements/truncation) in the *ERBB2‐*amplified group. The difference in frequency of pathogenic changes was not significant (Figure 4).

#### Number of gene mutations

We found 129 genes that exhibited mutations in the 20 patients but the distributions of the pathogenic mutations in these 129 genes were similar in both groups (83 versus 86), without statistical significance. We used the www.genecards.org site to double‐check the biological significance of the detected mutations. The highest numbers of gene mutations were detected in the *CDH1* gene (9 of 10 cases in the *ERBB2‐*unamplified group and 7 of 10 cases in the *ERBB2‐*amplified group) confirming lobular differentiation, but without statistical significance. The mutations/losses on chromosome 16 were as follows: *ERBB2‐*unamplified cohort: T748fs*23, Q610*, D687fs*35, F486fs*37, R74*, R54fs*5, splice site 48+2_48+17del16, E497*, and I415fs*2. HER2‐positive cohort: loss, Y797fs*19, F730fs*17, Q765*, H97fs*20, G212fs*2, and E689fs*34.

*ERBB2* gene sequence mutations were identified in 6 of 10 cases. Mutations in the *ERBB2‐*unamplified cohort on chromosome 17 were as follows: V777L, T27P, L755S, V777L, G366A, and P285S. There were no *ERBB2* sequence mutations in the *ERBB2‐*amplified cohort. This difference was statistically significant (*n* = 0.027).

Mutations in other genes were not significantly different in *ERBB2‐*amplified and *ERBB2*‐unamplified groups.

#### Frequency of gene amplifications

There were in total considerably more gene amplifications in the *ERBB2*‐amplified group (*n* = 73) than in the *ERBB2*‐unamplified group (*n* = 15); this difference was statistically significant (*p* = 0.009). All 10 of the *ERBB2*‐amplified cases featured ERBB2 copy numbers of 5 or greater, whereas none of the 10 *ERBB2*‐unamplified cases featured *ERBB2* copy numbers of 5 or greater. ERBB2 copy number alteration (CNA) exon ratio in the *ERBB2*‐amplified cases was as follows: 3.10, 3.20, 3.63, 4.2, 6.28, 7.06, 7.89, 8.34, 12.12, and 20.54. This difference was statistically significant (*p* = 0.006).

Seven of 10 *ERBB2*‐amplified patients featured coamplification of the *CDK12* gene. None of the *ERBB2*‐unamplified patients had *CDK12* amplifications. This difference was also statistically significant (*p* = 0.018). The following CNA exon ratios for *CDK12* were found: 2.79, 3.36, 3.58, 3.73, 4.06, 4.63, and 10.93.

Amplifications in other genes were not significantly different in the *ERBB2*‐amplified and *ERBB2*‐unamplified groups.

#### Number of gene copy losses

Gene copy losses were very rare events; only two cases in each group had detectable copy losses: *CUL4A* (ratio 0.47) and *RB1* (ratio 0.37) in the *ERBB2*‐unamplified group, and *CDH1* (ratio 0.22) and *MSTR1* (ratio 0.68) in the *ERBB2*‐amplified group. These differences were also not significant.

#### Number of gene rearrangements/truncations

Five patients in the *ERBB2*‐amplified group had rearrangements (*n* = 2) and truncations (*n* = 3); rearrangement in one patient on the *ERB2* gene (position 1: chr17:37872406–37872610 and position 2: chr17:37875897‐37876238) and in a second patient on the *PDGFRA* gene (position 1: chr4:55141126–55141218 and position 2: chr16:73556403–73556497). Truncation in one patient on the *CDK12* gene (position 1: chr17:37648999–37649185 and position 2: chr17:38481240–38481419), a second patient on the *BRCA1* gene (position 1: chr17:41243978–41244233 and position 2: chr17:28449439–28449657), and a third patient on the *NF1* gene (position 1: Chr17:29490272–29490518, position 2: Chr17:54467136–54467433). There was one patient in the *ERBB2*‐unamplified group with truncation on the *BRCA1* gene (position 1: chr17:41247768–41248053 and position 2: chr17:34211688–34211991). These differences were statistically not significant.

**Figure 5:**
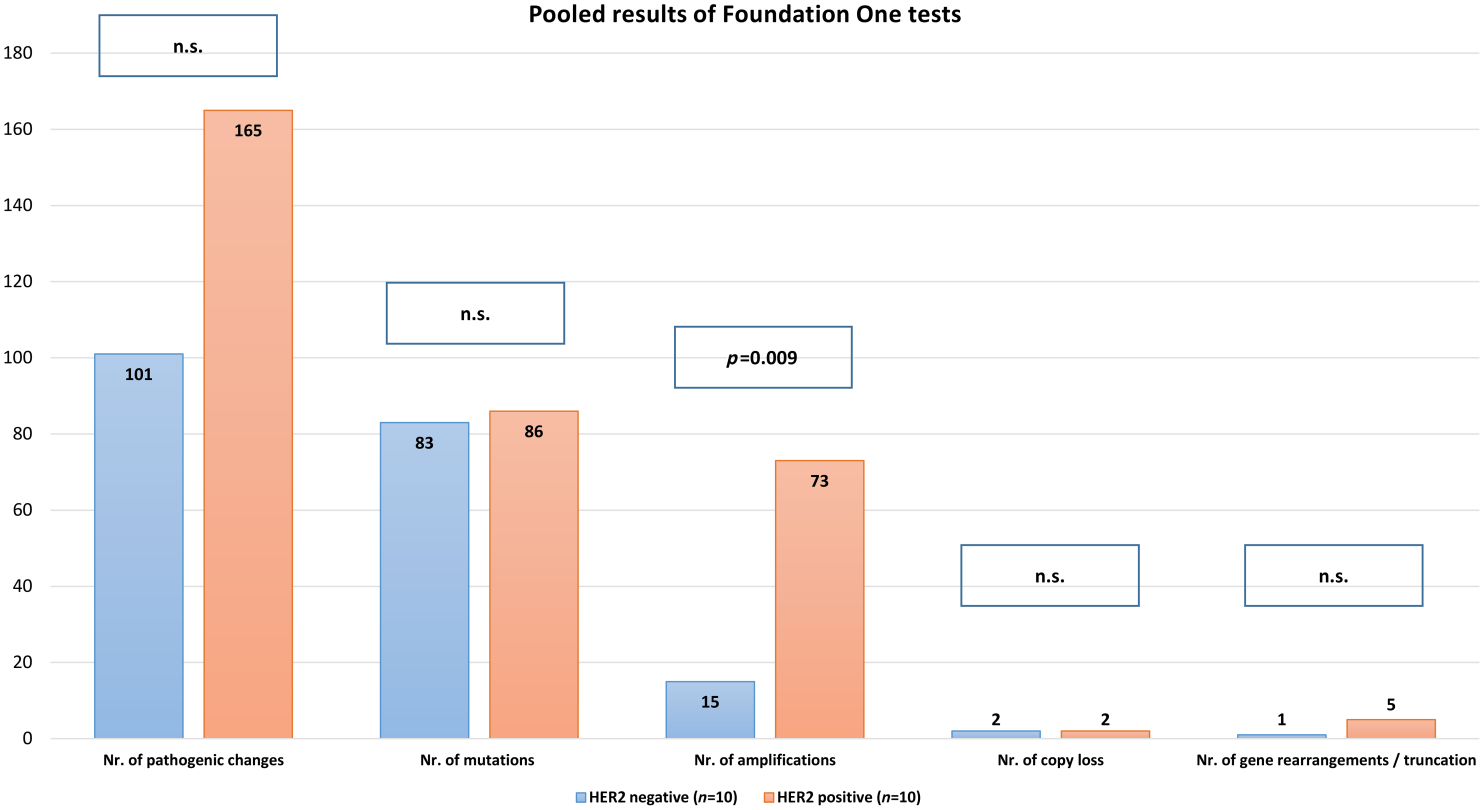
Graphical presentation of pooled FoundationOne®CDx test results comparing HER2‐positive (*n* = 10) and HER2‐negative (*n* = 10) breast carcinomas. The relative proportion (percentage) of gene amplification was significantly higher in HER2 positive than in HER2‐negative lobular carcinomas (*p* = 0.0009). Differences in the whole numbers of pathogenic changes, mutations, gene copy losses, and rearrangements/truncations were statistically not significant.

Mutation status, amplification status, copy loss, and re‐arrangement/truncation of both cohorts are shown in detail in supplementary material, Table S2, and Figure 5.

**Figure 6:**
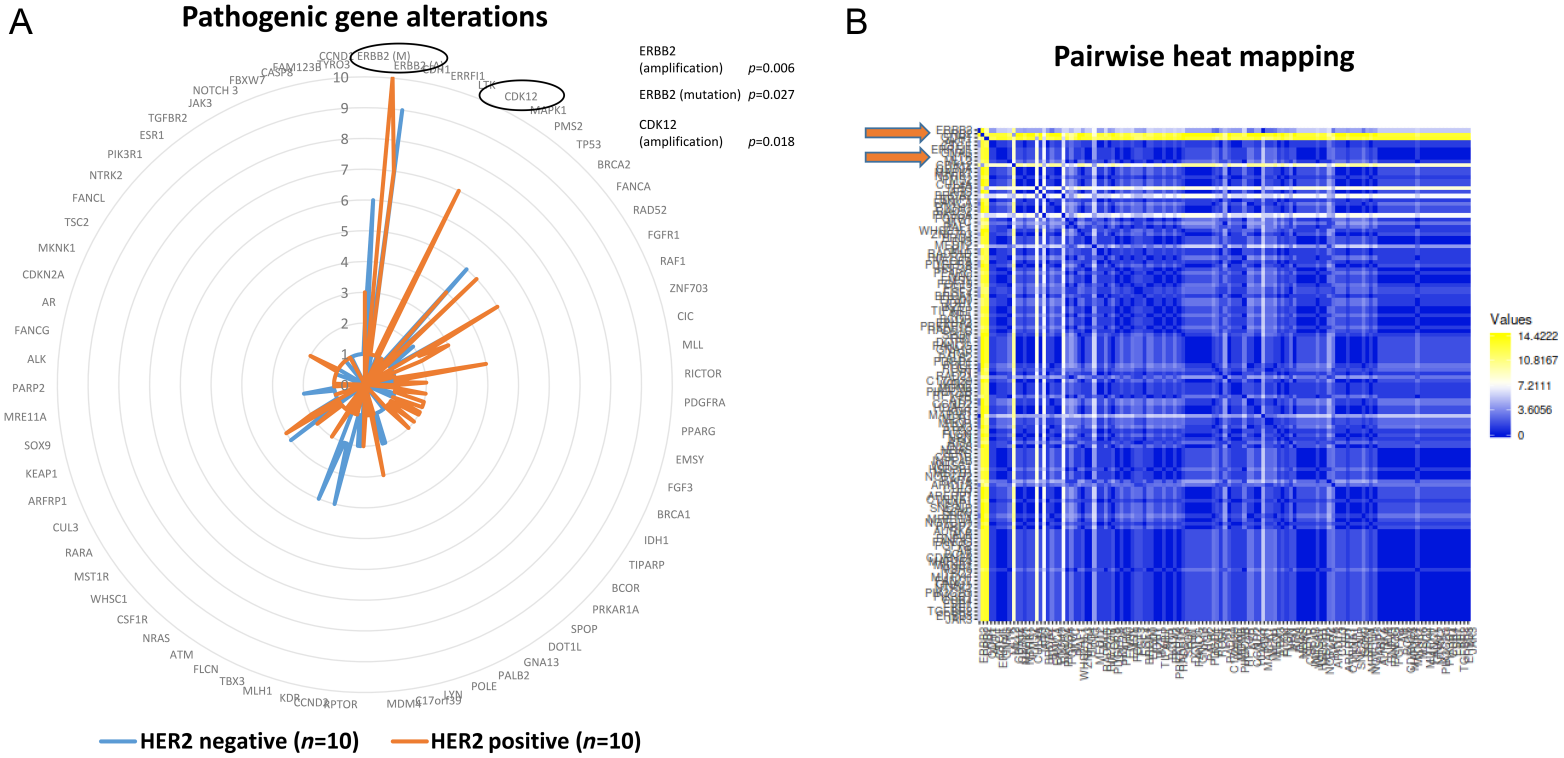
Graphical illustration of pathogenic mutations/amplifications detected *via* Foundation One tests in HER2‐positive and HER2‐negative breast cancer. (A) The complete list of pathogenic mutations is shown in net‐graphics. The scale gives the number of cases exhibiting pathogenic mutations and/or gene amplification. The *HER2* gene (named as *ERBB2*) and the *CDK12* gene have significant higher numbers of amplified cases in the HER2‐positive subgroup (*p* = 0.006 and *p* = 0.018, respectively) and ERBB2 mutations are significantly more common in the HER2‐negative subgroup (*p* = 0.0027). (B) The same dataset showing pairwise correlation with a heat mapping graphic. The upper lines (arrows to yellow as *ERBB2*, thick second white line as *CDK12*) correspond to the significantly expressed gene alterations in the HER2‐positive subgroup.

Correlations of genetic changes in a pairwise manner are illustrated in Figure 6A,B.

#### Tumor mutational burden

Low, intermediate, and high TMBs were evenly distributed in both groups without statistical difference.

#### Microsatellite instability

All 20 analyzed cases were microsatellite stable.

#### Loss of heterozygosity

All 20 cases had LOH, which was evenly distributed among the groups without any statistical significance. Graphical illustration of the results was carried out using Microsoft Excel 2023 and by the online toll Pairwise Comparison (www.heatmapper.ca).

Raw data for TMB, MSI, and LOH status are shown in Table 3 and are graphically illustrated in Figure 4A–C.

**Table 3:** Results of FoundationOne®CDx tests: (A) tumor mutational burden, (B) microsatellite instability, (C) loss of heterozygosity, and (D) summary of type of genetic alterations

(A) Tumor mutational burden using a three‐tiered scoring scheme (low–intermediate–high)			
Tumor mutational burden	HER2 negative (*n* = 8)	HER2 positive (*n* = 10)	*p* value
Low	7	7	ns
Intermediate	2	3	
High	1	0	

(B) Microsatellite instability using a two‐tiered scheme (stable versus instable)			
Microsatellite instability	HER2 negative (*n* = 8)	HER2 positive (*n* = 10)	*p* value
Stable	10	10	n.s.
Instable	0	0	

(C) Loss of heterozygosity (percentage)				
Loss of heterozygosity (percentage)	HER2 negative	Loss of heterozygosity (percentage)	HER2 positive	*p* value
Case 1	9.60%	Case 11	20.80%	n.s.
Case 2	2.90%	Case 12	7.00%	
Case 3	7.50%	Case 13	8.00%	
Case 4	15.80%	Case 14	8.30%	
Case 5	5.90%	Case 15	10.40%	
Case 6	19.40%	Case 16	26.00%	
Case 7	4.60%	Case 17	6.80%	
Case 8	3.20%	Case 18	8.20%	
Case 9	6.00%	Case 19	0.30%	
Case 10	10.00%	Case 20	6.60%	

(D) Summary of type of genetic alterations such as number of genetic changes, mutations, amplifications, copy‐loss, rearrangements, and truncations			
Summary of results Foundation One tests	HER2 neg (*n* = 10)	HER2 pos (*n* = 10)	*p* value
Nr. of pathogenic changes	101	165	ns
Nr. of mutations	83	86	ns
Nr. of amplifications	15	73	**0.009**
Nr. of copy loss	2	2	ns
Nr. of gene rearrangements/truncation	1	5	ns

Bold font indicates *p* < 0.05.

The 20 cases that underwent FoundationOne®CDx testing are shown with individual results in Table 4 (ERBB2‐unamplified cohort) and Table 5 (*ERBB2*‐amplified cohort).

**Table 4:** FoundationOne®CDx tests with clinicopathological parameters for the HER2‐negative cohort

*n* = 10	Case 1	Case 2	Case 3	Case 4	Case 5	Case 6	Case 7	Case 8	Case 9	Case 10
HER2‐negative ILC										
Histological subtype	Classical lobular	Classical lobular	Pleomorphic lobular	Pleomorphic	Classical lobular	Classical lobular	Classical lobular	Classical lobular	Classical lobular	Classical lobular
ER (in %)	100	90	30	10	60	70	2	100	100	100
PR (in %)	90	90	Negative	Negative	5	2	Negative	100	80	80
HER2 IHC	Score 0	Score 0	NA	Score 1+	Score 0	Score 2+	Score 0	Score 1+	Score 2+	Score 1+
HER2 FISH	Negative	Negative	Negative	Negative	NA	Negative	Negative	Negative	Negative	Negative
Grade	2	2	3	3	2	2	2	2	2	2
pT	pT1c	pT1c	NA	pT3	NA	pT4	NA	pT1b (m)	NA	pT3
pM	NA	NA	pM1 (hepatic)	pM1 (cerebral)	pM1 (hepatic)	pM1 (pleural)	pM1 (bone, visceral)	pM0	pM1 (visceral)	NA
pN	pN0	pN0	NA	pN1	NA	NA	NA	pN0	pN1	pN1
Age (years)	73	55	61	72	74	80	62	71	65	73
CCND1 amplifications	No	No	No	No	No	No	Ratio 1.64	No	No	ratio > 7
ERBB2 mutations	V777L	T27P	L755S	No	No	No	No	V777L	G366A	P285S
ERBBB2 amplification	No	No	No	No	No	No	No	No	No	No
CDH1 mutations	T748fs*23	Q610*	D687fs*35	F486fs*37	R74*	No	R54fs*5	Splice site 48 + 2_48 + 17del16	E497*	I415fs*2
CDK12 amplifications	No	No	No	No	No	No	No	No	No	No
Other mutations	CD79a, MAPK1, NTRK1, PMS2	TP53, APC, BRCA2, BRIP1, FANCA, PTCH1, RAD52, SPEN	AKT1, TP53, DOT1L, FANCC, GNA13, HGF, PALB2, PDCD1, POLE, ROS1	TP53, FANCA, PIK3CA, ATR, CCDN2, HDAC1, KDR, MAP3K1, MLH1, MTOR, TBX3	TP53, GNAS, PIK3CA (2x), MLL, ATR, MAP3K1, ATM, DIS3	BRCA2, RADS1D, ROS1, MAP3K1, MTOR, MST1R	CTCF, TP53, PIK3CA, ZNF703, PDGFRA, C17orf39, CCDN2, MLH1, TBX3, MST1R, ARID1A, CUL3, FH, ARFRP1, CTNNA1, KEAP1, SOX9, SPEN	VGFA, BRCA1, NAP3K1, ARIDA1, NOTVH1, PARP2	CASP8, CDKN2A,ERB4, ESR1, FAM123B, MLL2, MST1R, NOTCH2,STK11, TBX3 (2x), TSC2, TYRO3	CHEK2, FBXW7, FOXL2, NOTCH3, SMAD4, TBX3
Amplification	No	No	No	MYC, LYN, RAD21, C17orf39, IKBKE, MDM4, PIK3C2B, RPTOR, BTG2, FLCN, NBN	No	N0	FGF3, FGF4, MRE11A	No	No	FGF19, FGF3, FGF4
Other gene loss	CUL4A	No	RB1	No	No	No	No	No	No	No
Other re‐arrangements /truncation	No	No	No	No	No	No	No	BRCA1	No	No
Mutational burden	2.52 Muts/Mb	3.78 Muts /Mb	3.78 Muts/mb	8.83 Muts/Mb	5.04 Muts/Mb	1.26 Muts/Mb	22.7 Muts/Mb	2.52 Muts/Mb	4 Muts/Mb	3 Muts/Mb
Microsatellite status	Stable	Stable	Stable	Stable	Stable	Stable	Stable	Stable	Stable	Stable
Loss of heterozygosity	9.60%	2.90%	7.50%	15.8%	5.9%	19.4%	4.6%	3.2%	6%	10%

ER, estrogen receptor; FISH, fluorescence *in situ* hybridization; IHC, immunohistochemistry; ILC, invasive lobular carcinoma; NA, not available; PR, progesterone receptor.

**Table 5:** FoundationOne®CDx tests with clinicopathological parameters for the HER2‐positive cohort

*n* = 10	Case 11	Case 12	Case 13	Case 14	Case 15	Case 16	Case 17	Case 18	Case 19	Case 20
HER2‐positive ILC										
Histological subtype	Pleomorphic lobular	Classical lobular	Pleomorphic lobular	Pleomorphic lobular	Classical lobular	Classical lobular	Classical lobular	Pleomorphic	Pleomorphic type	Pleomorphic type
ER (in %)	20	80	100	100	80	Negative	90	Negative	Negative	80
PR (in %)	20	5	15	100	1	Negative	90	Negative	Negative	80
HER2 IHC	NA	Score 3+	Score 3+	Score 3+	Score 3+	Score 3+	NA	NA	NA	score 3+
HER2 FISH	Amplified	Amplified	Amplified	Amplified	Amplified	Amplified	Amplified	Amplified	Amplified	Amplified
Grade	3	2	3	2	2	3	2	3	3	2
pT	pT3	pT2	pT3	pT1c	pT2	pT4	pT3	pT1c	cT4	NA
pM	pM1 (visceral)	pM0	pM1 (hepatic)	pM0	pM0	pM0	pM0	pM0	cM0	pM1 (pleura)
pN	NA	pN0	pN1	pN0	pN1	pN3	pN3	pN1	cN2	pN1
Age (years)	56	72	49	55	63	46	63	43	55	69
CCND1 amplifications	No	Ratio 2.16	No	Ratio 5.21	Ratio 2.06	No	No	No	No	No
ERBB2 mutations	No	No	No	No	No	No	No	No	No	No
ERBBB2 amplification	Ratio 7.06	Ratio 3.20	Ratio 7.89	Ratio 4.2	Ratio 12.12	Ratio 6.28	Ratio 20.54	Ratio 8.34	Ratio 3.10	Ratio 3.63
CDH1 mutations	Loss	Y797fs*19	F730fs*17	Q765*	No	No	H97fs*20	No	G212fs*25	E689fs*34
CDK12 amplifications	No	No	No	Ratio 3.73	Ratio 10.93	Ratio 4.63	Ratio 4.06	Ratio 3.58	Ratio 3.36	Ratio 2.79
Other mutations	TP53, PIK3CA, BRD4, CIC, MED12, MLL, RAD51D, RICTOR, VEGFA	MLL2, PIK3CA, BRCA1, EP300, IDH1, IKZF1	PIK3CA, TIPARP, BCOR, EPHA3	AKT1, CTCF, ERFl1, GNAS, LTK, MLL2, BRCA2	BRIP1, PIK3CA, MED12, RPTOR, ATM, ALK, RNF43, RPTOR	TP53, MAP3K1, ATM, BRCA2, BRIP1, PIK3CA, MED12, RPTOR, MAP3K1, ATM, CTNNA1, ALK, AR, BCL6, CDKN2A, MAP2K4, MKNK1, MSH6, TSC2	PIK3CA, MED12, RICTOR, ROS1, MTOR, WHSC1, ARID1A, MUTYH, FANCL, GNA11, NTRK1, NTRK2, PIK3C2G	TP53, BRCA2, MED12, NF1, MSH6, PIK3R1, CDK4, ESR1, KEL, TGFBR2	ROS1, ARID1A, NOTCH1, ERBB3, JAK3	TP53, EP300, NF1, PIK3C2B, CSF1R, INPP4B, WHSC1
Amplification	FGFR1, MYC, RAF1, WHSC1L1, ZNF703, H3F3A, PPARG, VHL	EMSY, FGF19, FGF3, FGF4	PRKAR1A, RAD51C, SPOP	No	GNAS, FGF19, FGF3, FGF4, PRKAR1A, SPOP, RAD21, RPTOR, ARFRP1, AURKA, FANCG	BRCA2, RAF1, PPARG, EPHA3, FGFR2	RARA	RPTOR	MYC, RAD21, RARA	FGFR1, MYC, WHSC1L1, EPHA3, LVN, RAD21, IKBKE, MDM4, PIK3C2B, BTG2, NBN, NRAS, TERC, HSD3B1, NOTCH2, RARA
Other gene loss	CDH1	No	No	N0	No	No	No	No	No	MST1R
Other re‐arrangements /truncation	PDGFRA	No	NF1	N0	No	No	ERBB2	No	No	RARA, BRCA1
Mutational burden	7.57 Muts/Mb	5.04 Muts/Mb	0 Muts/Mb	1.26 Muts/Mb	2.52 Muts/Mb	3.77 Muts/Mb	5.04 Muts/Mb	2.52 Muts/Mb	0.0 Muts/Mb	0 Muts/Mb
Microsatellite status	Stable	Stable	Stable	Stable	Stable	Stable	Stable	Stable	Stable	Stable
Loss of heterozygosity	20.8%	7.0%	8.00%	8.3%	10.40%	26.00%	6.80%	8.20%	0.30%	6.60%

ER, estrogen receptor; FISH, fluorescence *in situ* hybridization; IHC, immunohistochemistry; ILC, invasive lobular carcinoma; NA, not available; PR, progesterone receptor.

### FMI insight database

In the entire FMI database of 463,546 cases, only 1.2% featured *CDK12* alterations of any type of which only 1.1% are amplifications (0.01%). The tumor types with *CDK12* amplification are most frequently prostate cancer, breast cancer (six cases, none are ILBC), and ovarian cancer. Of the small number of *CDK12*‐amplified tumors, 21% have coamplification of the CCND1/FGF3, FGF4, amplification; 17% have *MYC* amplification, and a number of other genes are coamplified. No *CDK12*‐amplified tumors have *ERBB2* coamplification.

Among 44,293 breast cancers in the FMI Insights database, *ERBB2* amplification is at 8% and *ERBB2* sequence mutation at 2.9%. The frequency of *CDK12* amplification is 0% and the *CDK12* mutation frequency is 1.3%.

In 2,502 ILBC (5.6% of total breast cancers), *ERBB2* amplification is 2.3%, ERBB2 sequence mutation is 9.2%, *CDK12* amplification is 0%, and the *CDK12* mutation frequency is 0.5%.

### Correlation of clinical parameters and HER2 status with survival data

Kaplan–Meier statistics confirmed a significant lower overall survival (OS) in higher tumor stage (pT2 and above) (*p* = 0.0096) and in nodal positive cases (*p* = 0.0028) especially within the first 120 months after diagnosis, also after adjusting for HER2‐status. Differences in long‐term follow‐up were similar between stage‐matched *ERBB2*‐unamplified and positive ILBC. HER2 status did not impact disease‐free survival (time to recurrence or distant metastases) in this cohort. Cox regression values were not significant for OS alone but showed a significant correlation between positive HER2 status and higher tumor stage or positive nodal status (supplementary material, Figure S1).

## Discussion

In this study, we demonstrate that HER2‐positive invasive lobular carcinoma represents a biologically distinct subgroup with poor prognostic features, which differs from HER2‐negative lobular carcinoma in terms of immunophenotype, clinical parameters, and molecular alterations. The underlying *CDK12*/*HER2* gene coamplification, which is substantially detectable in our HER2‐positive lobular breast carcinoma cohort, is a characteristic molecular phenomenon in this subgroup, possibly contributing to poor prognostic behavior.

According to our knowledge and most current literature data, this is the first study to provide comprehensive molecular data on HER2‐positive invasive lobular carcinoma and demonstrating that *CDK12/HER2* gene coamplification is a common underlying mechanism in this subgroup.

Invasive lobular carcinomas most often represent a hormone receptor‐positive, *ERBB2*‐unamplified luminal intrinsic subtype that accounts for 10–15% of all breast carcinomas [1, 2, 3]. *ERBB2* amplification is a rare phenomenon in lobular breast carcinoma and sparse literature data point to poorer biological properties and unfavorable prognostic features. However, the data are not clear on the underlying mechanism resulting in the unfavorable biology [3, 4, 5, 6, 7, 8]. In a series of nine cases, He *et al* found that HER2‐positive ILBC occurs at a younger age and is more often hormone receptor negative and nodal positive [4]. Zhang *et al* showed in a series of 21 nonpleomorphic HER2‐positive ILBCs that patients are more often hormone receptor negative and have lymphovascular invasion; however, in this series, patients were older than those with HER2‐negative ILBCs [5]. In our study, clinic‐pathological data corroborate these observations. We also identified larger tumor and nodal stage, higher grade, and hormone receptor negativity similarly to the reported small series. However, in our series, patients were only slightly younger than *ERBB2*‐unamplified ILBC patients. Interestingly, we analyzed the expression of a large panel of immunohistochemical markers and only SOX10 was more frequently expressed in *ERBB2*‐amplified ILBC, a marker that is typically positive in triple‐negative NST and non‐NST carcinomas. All other markers resulted in no difference in association with *ERBB2*‐amplified status [1].

Regarding prognosis and therapy responses, sparse data are available on HER2‐positive ILBCs. In 2022, Okina *et al* reported that HER2‐positive ILBC especially in advanced stage had worse prognosis and shortened OS than HER2‐positive NST carcinomas and postulated the inconsistent administration of anti‐HER2 agents as a possible explanation [6]. A similar observation was reported in a recent French study, which found that only HER2‐positive invasive ductal carcinomas responded to targeted anti‐HER2 therapy, and this effect was not seen in HER2‐positive lobular carcinoma [3]. Da Ros on the contrary did not find differences in recurrence rate between HER2‐positive ILBC and NST carcinomas, but showed a broader variation in mutational landscape in ILBC than in NST, postulating the more frequent molecular alterations as the leading cause for worse prognosis [8]. In our series, all *ERBB2*‐amplified patients received anti‐HER2 therapy, and there were more distant metastases in this group compared with ERBB2‐unamplified patients in the follow‐up period. However, therapy regimens that also included adjuvant chemotherapy were not identical in our cohort possibly explaining the lack of survival correlation to distant metastases. Differences in long‐term follow‐up did not, however, show significant differences, which is most likely explained by the different therapy regimens in the otherwise rather heterogeneous cohort and the low number of rare *ERBB2*‐amplified subset of ILBC.

Despite a trend toward shorter OS in *ERBB2*‐amplified ILBC (119.6 ± 15.6 months) versus ERBB2‐unamplified ILBC (149.9 ± 6.6 months), Kaplan–Meier survival analysis did not show a statistically significant difference between HER2‐positive and HER2‐negative ILBC, also when only higher tumor stage or positive nodal stage were studied. Independent of these results, HER2‐positive ILBCs were more frequently associated with prognostically unfavorable characteristics such as higher grade, higher tumor stage, and lymph node positivity compared with HER2‐negative ILBC.

Molecular alterations in most lobular breast carcinomas involve somatic genetic alterations in the *CDH1* gene locus on 16q22.1 resulting in loss of function of the *CDH1* gene, loss of IHC staining for E‐cadherin protein, and typical dyscohesive spread of invasive lobular carcinoma cells [1, 7, 22]. This molecular phenomenon together with further alterations of the catenin adherens complex (such as alfa‐catenin, beta‐catenin, and catenin p120) has become a useful diagnostic tool in the histopathological diagnosis of invasive lobular carcinoma [1, 7, 22]. This phenomenon was also observed in some precursor HER2‐positive LCIS lesions, possible pointing to stepwise evolution of HER2‐positive ILC along with *CDK12* amplification. Membranous loss of the E‐Cadherin and the catenin complex detected by IHC with corresponding dissolute cellular morphology helps to distinguish lobular breast carcinoma from NST carcinoma and supports the diagnosis of lobular differentiation in about 85% of ILBC [1, 7, 22]. In line with the current WHO guidelines, the histological diagnosis of invasive lobular carcinoma is based on H&E morphology and on the immunohistochemical characterization of E‐cadherin and the catenin complex independently from the presence of underlying *CDH1* gene mutations [1, 7, 22]. *CDH1* gene mutation, even though frequent in ILBC, does not, according to current guidelines, *per se* define or exclude lobular differentiation alone and molecular *CDH1* status should be interpreted only together with the morphology [1, 7, 22].

In our series, next‐generation sequencing analyses identified *CHD1* mutations or copy loss in 80% of the cases that underwent molecular testing, further confirming lobular differentiation. Regarding *ERBB2* gene alterations in ILBC, it is of note that *ERBB2* sequence mutations were detected only in *ERBB2* unamplified, mostly classical ILBC (in 6 of 10 cases), in some cases with identical mutations (V777L) as described previously by Christgen *et al* [7]. Ross *et al* also reported a higher frequency of *ERBB*2 gene mutations in *ERBB2*‐unamplified invasive lobular carcinomas [23]. On the contrary, no *ERBB2* sequence mutations were detectable in *ERBB2*‐amplified ILBC, this study group had *HER2* gene amplification in all cases. *ERBB2* sequence mutations in ILBC are often found in metastatic and local recurrence biopsies and this reflects the impact of hormonal therapies, especially aromatase inhibitors and are considered acquired resistance mutations (especially in cases where acquired *ESR1* mutations are not identified) [3, 22].

In this study, we report *CDK12* gene amplification in 7 of 10 *ERBB2*‐amplified ILBCs. No *CDK12* gene amplifications were reported in 44,293 invasive breast carcinomas. In the entire FMI database of 463,546 tumors of all types, only 0.01% have *CDK12* amplifications.

Therefore, *CDK12* amplification is an extremely rare event in cancer, whereas *CDK12* amplification is not infrequent in *ERBB2*‐amplified ILBC. Interestingly, we have determined that, in the rare subgroup of *ERBB2*‐amplified ILBC, 70% of them had concomitant amplification of the *CDK12* gene. CDK12 (cyclin‐dependent kinase 12) has been recently discovered as a therapeutic target as CDK12 can induce DNA damage repair [22, 24, 25, 26]. In breast cancer, concurrent amplification of the *CDK12* and *ERBB2* genes has been described in ductal type NST carcinomas and in association with poor prognosis, worse response to anti‐HER2 treatment and also resistance to endocrine therapy [24, 25, 26, 27, 28]. Patients without concomitant *CDK12* amplification in HER2‐positive NST carcinomas had better prognosis [24, 25, 26, 27, 28]. We are the first to show that *ERBB2*‐amplified invasive lobular carcinomas similarly to *ERBB2*‐amplified NST breast carcinomas do exhibit concomitant *CDK12*/*ERBB2* gene amplification. This phenomenon likely explains why *ERBB2*‐amplified ILBC behaves in a more similar clinical manner to *ERBB2*‐amplified NST carcinomas as has been already postulated in sparse previous literature sources on NST breast carcinomas [3, 4, 5, 6, 29]. However, it is of note that therapy failure is best addressed in a neoadjuvant setting, which in our series has not been the case and should be potentially addressed in future studies.

The lower frequency of ILBC in FMI testing compared to the textbook incidence may represent an overall better prognosis of ILBC than IDC and lower numbers of cases sent for sequencing. Further, one might hypothesize that the low incidence of *CDK12* amplification in the whole FMI database most likely represents the fact that *ERBB2*‐amplified breast cancer especially lobular types might not have been submitted to FMI testing as such cases undergo primarily HER2‐directed therapy regimens. On the other hand, only FMI‐detected HER2 positivity remains unclear in terms of ‘real’ HER2‐positive status as the FMI databank lacks information on reflex testing via HER2 IHC and/or ISH analyses. Without the currently required routine HER2 testing, which is IHC and/or ISH, it is not possible to draw any solid conclusion on the low HER2 positivity without *CDK12* amplification measured only by molecular profiling.

It has also been shown that dual blockage of CDK12/HER2 in HER2‐positive NST breast cancer results in reduced endocrine resistance and better response to anti‐HER2 therapy also in the metastatic setting [24, 25, 26, 27, 28]. In patient‐derived organoids *in vitro* or in xenografts *in vivo*, CDK12 inhibition through the PI3K/AKT pathway combined with Lapatinib reduced breast cancer progression [27]. It is, therefore, reasonable to assume that higher level of CDK12 expression/gene amplification can serve as a prognostic marker not only in NST but also in *ERBB2*‐amplified ILBC. Oncologists should therefore be aware of this gene expression association in *ERBB2*‐amplified ILBC [24, 25, 26, 27, 28].

In conclusion, our study shows evidence that *ERBB2*‐amplified ILBC represents a distinct subgroup of lobular carcinomas with unfavorable prognostic characteristics and a distinct molecular genetic phenotype. Higher histological grade, higher tumor stage, and more frequent nodal involvement significantly distinguish *ERBB2*‐amplified ILBC from *ERBB2*‐unamplified ILBC. Mutations in the *ERBB2* gene occur in *ERBB2*‐unamplified ILBC, whereas coamplification of the *CDK12* and *ERBB2* genes are characteristic features of *ERBB2*‐amplified ILBC. Coamplification of the *CDK12* and *ERBB2* genes has previously only been reported in NST breast carcinomas; this study reports this finding for the first time in lobular breast carcinoma. This association can be potentially considered in therapeutic settings to reduce poorer response to anti‐HER2 treatment and to resistance to endocrine therapy.

## Author contributions statement

MSF contributed to patient collection, cohort compilation, TMA construction, analysis and interpretation of TMA and FMI data, and drafting the manuscript. MZ supervised FMI data retrieval, interpreted FMI results and critically read the manuscript. BP, IW, HF and CT interpreted clinical aspects and study results, and critically read the manuscript. EIS supervised follow‐up data retrieval and conducted statistical analyses, interpreted survival data and critically read the manuscript. JH retrieved clinical follow‐up data and critically read the manuscript. JR supervised FMI data retrieval and interpretation of results, and critically read the manuscript. HM supervised and interpreted FMI data, and drafted and finalized the manuscript. ZV developed the study design and supervised cohort compilation, TMA and FMI data interpretation, drafting and finalizing the manuscript.

## Supporting information


**Figure S1.** Kaplan–Meier curves for overall survival compared between different tumor stages and nodal status in the whole cohort and adjusted for HER2 status in invasive lobular carcinomas
**Table S1.** Laboratory data for the immunohistochemical stains
**Table S2.** FoundationOne®CDx mutations/amplifications

## Data Availability

All raw data are available upon request.
