# Supplementary material for: ERBB2‐amplified lobular breast carcinoma exhibits concomitant CDK12 co‐amplification associated with poor prognostic features
Source: J Pathol Clin Res. 2024 Jan 21;10(2):e12362. doi: 10.1002/2056-4538.12362 (PMC10800294; doi:10.1002/2056-4538.12362)
Supplement: Supplementary file 1 — Figure S1. Kaplan–Meier curves for overall survival compared between different tumor stages and nodal status in the whole cohort and adjusted for HER2 status in invasive lobular carcinomas Table S1. Laboratory data for the immunohistochemical stains Table S2. FoundationOne®CDx mutations/amplifications [file CJP2-10-e12362-s001.pdf]

ERBB2-amplified lobular breast carcinoma exhibits concomitant *CDK12* co-amplification associated with poor prognostic features

M Foster-Sack *et al.*, *J Pathol Clin Res*, https://doi.org/10.1002/2056-4538.12362

Supplementary Figure S1
Supplementary Tables S1 and S2

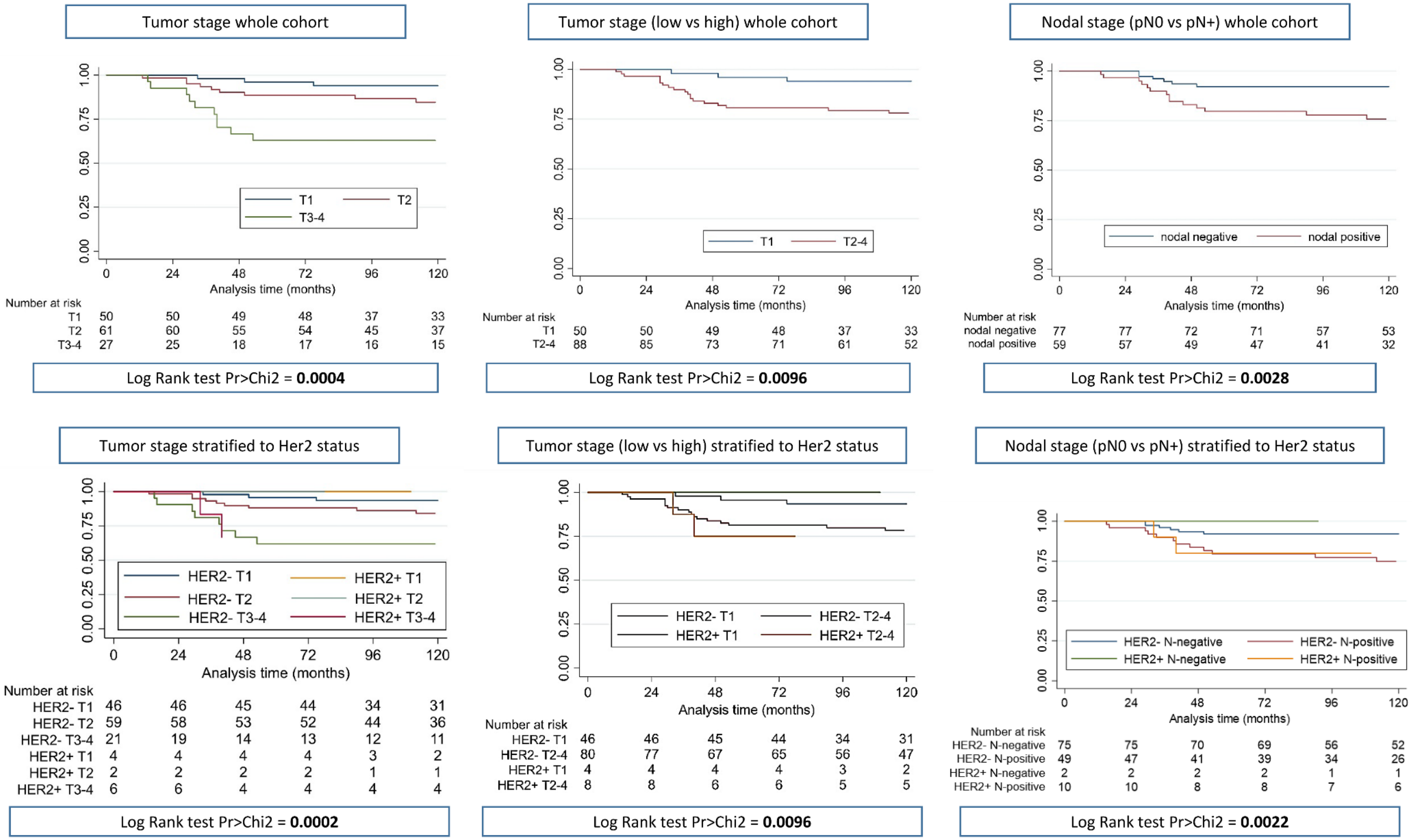

Figure S1. Kaplan Meier curves for overall survival compared between different tumor stages and nodal status in the whole cohort and adjusted for Her2 status in invasive lobular carcinomas

Table S1. Laboratory data for the immunohistochemical stains

Characteristics of immunohistochemical stains						
**Protein**	**Clone**	**Dilution**	**Pretreatment**	**Visualization**	**Staining machine**	**Producer**
Estrogen receptor	SP1	Prediluted	CC1 m	UltraView	Ventana Autostainer	Ventana-Roche
Progesterone receptor	1E2	Prediluted	CC1 52	UltraView	Ventana Autostainer	Ventana-Roche
HER2	4B5	Prediluted	CC1 m	UltraView	Ventana Autostainer	Ventana-Roche
Ki-67 (MIB-1)	30-9	Prediluted	CC1 32	UltraMap Rabbit	Ventana Autostainer	Cell Marque
E-Cadherin	monoclonal NCH-38	1:100	CC1 40	OptiView	Ventana Autostainer	DAKO
Catenin p120	monoclonal 98	1:200	CC1 48	OptiView	Ventana Autostainer	BD Biosciences
CK 5/6	D5	Prediluted	CC1 48	OptiView	Ventana Autostainer	Ventana
p63	4A4	Prediluted	CC1 64	OptiView	Ventana Autostainer	Ventana
PTEN	monoclonal 138G6	1:200	Bond H2	Refine HRP	Leica Bond Autostainer	Cell Signaling Technology
PIK3CA	Sigma HPA009985 polyclonal	1:25	CC1 standard	UltraMap Rabbit	Ventana Autostainer	Sigma Chemical Company

p53	DO7	1:80	CC1 32	OptiView	Ventana Autostainer	DAKO
mTOR	49F9	1:100	CC1 92	OptiView	Ventana Autostainer	Cell Signaling Technology
SOX2	EPR3131	1:100	CC1 92	OptiView	Ventana Autostainer	Epitomics, Inc./abcam
SOX9	EP317	1:400	CC1 48	OptiView	Ventana Autostainer	Epitomics, Inc./abcam
SOX10	BC34	1:150	Bond H2	Refine HRP	Leica Bond Autostainer	Biocare Medical
SLUG	C19G7	1:25	CC1 standard	UltraMap Rabbit	Ventana Autostainer	Cell Signaling Technology
CD44	G44-26	1:100	CC1 8	OptiView	Ventana Autostainer	PharMingen (Becton Dickinson)
TWIST	Abcam ab 56581	1:200	CC1 mild	UltraMap Rabbit	Ventana Autostainer	Abcam Limited
GATA3	monoclonal L50-823	1:200	CC1 64	OptiView	Ventana Autostainer	Cell Marque Lifescreen Ltd.
brst-2 (GCDFP-15)	monoclonal D6	1:4	CC1 24	OptiView	Ventana Autostainer	Covance / BioLegend
CK7	SP52	Prediluted	P1 (4)	OptiView	Ventana Autostainer	Abcam Limited
NY-BR-1	polyclonal	1:2500	Bond H2 (30)	Refine HRP	Leica Bond Autostainer	LS Bio
Mammaglobin	monoclonal 304-1A5	1:200	CC1 16	OptiView	Ventana Autostainer	DAKO

Table S2. FoundationOne®CDx mutations/amplifications

**Gene mutations /amplifications** cursiv: not pathogenic, bold: pathogenic	Her2 neg n=10	Her2 pos n=10	p=
**CCND1** CNA-COPY-Number (amplification)	2	3	ns
**ERBB2**			
SV-Protein change (mutation)	6	0	**0.027**
CNA-COPY-Number (amplification)	0	10	**0.006**
**CDH1**			
SV-Protein-Change (mutation)	9	7	ns
CNA-COPY-Number (loss)	0	1	ns
**AKT1**			
SV-Protein-Change (mutation)	1	1	ns
**CTCF**			
SV-Protein-Change (mutation)	1	1	ns
**ERRFI1**			
SV-Protein-Change (mutation)	0	1	ns
**GNAS**			
CNA-COPY-Number (amplification)	0	1	ns

Gene mutations /amplifications cursiv: not pathogenic, bold: pathogenic	Her2 neg n=10	Her2 pos n=10	p=
**MLL** SV-Protein-Change (mutation)	1	1	ns
**RAD51D**			
SV-Protein-Change (mutation)	1	1	ns
**RICTOR**			
SV-Protein-Change (mutation)	0	2	ns
**VEGFA**			
SV-Protein-Change (mutation)	1	1	ns
**PDGFRA**			
SV-Protein-Change (mutation)	1	0	ns
REARR-GENE2 (rearrangement)	0	1	ns
**H3F3A**			
CNA-COPY-Number (amplification)	0	1	ns
**PPARG** CNA-COPY-Number (amplification)	0	2	ns
**VHL**			

Gene mutations /amplifications cursiv: not pathogenic, bold: pathogenic	Her2 neg n=10	Her2 pos n=10	p=
**LYN** CNA-COPY-Number (amplification)	1	1	ns
**RAD21**			
CNA-COPY-Number (amplification)	1	3	ns
**C17orf39**			
SV-Protein-Change (mutation)	1	0	ns
CNA-COPY-Number (amplification)	1	0	ns
**IKBKE** CNA-COPY-Number (amplification)	1	1	ns
**MDM4** CNA-COPY-Number (amplification)	1	1	ns
**PIK3C2B** CNA-COPY-Number (amplification)	1	1	ns
**RPTOR** SV-Protein-Change (mutation)	0	2	ns
CNA-COPY-Number (amplification)	1	3	ns

Gene mutations /amplifications cursiv: not pathogenic, bold: pathogenic	Her2 neg n=10	Her2 pos n=10	p`=
**ARFRP1** SV-Protein-Change (mutation)	1	0	ns
CNA-COPY-Number (amplification)	0	1	ns
**CTNNA1**			
SV-Protein-Change (mutation)	1	1	ns
**KEAP1**			
SV-Protein-Change (mutation)	1	0	ns
**SNCAIP** SV-Protein-Change (mutation)	1	0	ns
**SOX9** SV-Protein-Change (mutation)	1	0	ns
**SPEN** SV-Protein-Change (mutation)	2	0	ns
**MRE11A** CNA-COPY-Number (amplification)	1	0	ns
**NOTCH1**			

**LTK**			
SV-Protein-Change (mutation)	0	1	ns
**MLL2**			
SV-Protein-Change (mutation)	1	2	ns
**CDK12**			
CNA-COPY-Number (amplification)	0	7	**0.018**
**CD79A**			
SV-Protein-Change (mutation)	1	0	ns
**MAPK1**			
SV-Protein-Change (mutation)	1	0	ns
**NTRK1**			
SV-Protein-Change (mutation)	1	1	ns
**PMS2**			
SV-Protein-Change (mutation)	1	0	ns
**CUL4A**			
CNA-COPY-Number (loss)	1	0	ns
**TP53**			
SV-Protein-Change (mutation)	5	4	ns
**APC**			
CNA-COPY-Number (amplification)	0	1	ns
**EMSY**			
CNA-COPY-Number (amplification)	0	1	ns
**FGF19**			
CNA-COPY-Number (amplification)	1	2	ns
**FGF3**			
CNA-COPY-Number (amplification)	1	2	ns
**FGF4**			
CNA-COPY-Number (amplification)	1	2	ns
**BRCA1**			
REARR-GENE2 (truncation)	1	1	ns
SV-Protein-Change (mutation)	0	1	ns
**EP300**			
SV-Protein-Change (mutation)	0	2	ns
**IDH1**			
SV-Protein-Change (mutation)	0	2	ns
**IKZF1**			
SV-Protein-Change (mutation)	0	1	ns
TIPARP			
**ATR**			
SV-Protein-Change (mutation)	2	0	ns
**CCND2**			
SV-Protein-Change (mutation)	2	0	ns
**HDAC1**			
SV-Protein-Change (mutation)	1	0	ns
**KDR**			
SV-Protein-Change (mutation)	1	0	ns
**MAP3K1**			
SV-Protein-Change (mutation)	4	1	ns
**MLH1**			
SV-Protein-Change (mutation)	2	1	ns
**MTOR**			
SV-Protein-Change (mutation)	2	1	ns
**TBX3**			
SV-Protein-Change (mutation)	4	0	ns
**BTG2**			
CNA-COPY-Number (amplification)	1	1	ns
**FLCN**			
SV-Protein-Change (mutation)	1	1	ns
**PARP2**			
SV-Protein-Change (mutation)	1	0	ns
**AURKA**			
CNA-COPY-Number (amplification)	0	1	ns
**ALK**			
SV-Protein-Change (mutation)	0	1	ns
**RNF43**			
SV-Protein-Change (mutation)	0	1	ns
**FANCG**			
CNA-COPY-Number (amplification)	0	1	ns
**FGFR2**			
CNA-COPY-Number (amplification)	0	1	ns
**AR**			
SV-Protein-Change (mutation)	0	1	ns
**BCL6**			
SV-Protein-Change (mutation)	0	1	ns
**CDKN2A**			
SV-Protein-Change (mutation)	1	1	ns

SV-Protein-Change (mutation)	1	0	ns
**BRCA2**			
SV-Protein-Change (mutation)	3	5	ns
CNA-COPY-Number (amplification)	0	1	ns
**BRIP1**			
SV-Protein-Change (mutation)	1	1	ns
**FANCA**			
SV-Protein-Change (mutation)	2	0	ns
**PTCH1**			
SV-Protein-Change (mutation)	1	0	ns
**RAD52**			
SV-Protein-Change (mutation)	1	0	ns
**PIK3CA**			
SV-Protein-Change (mutation)	3	5	ns
**FGFR1**			
CNA-COPY-Number (amplification)	0	2	ns
**MYC**			
CNA-COPY-Number (amplification)	1	3	ns
**RAF1**			

SV-Protein-Change (mutation)	0	1	ns
**NF1**			
SV-Protein-Change (mutation)	0	2	ns
REARR-GENE2 (truncation)	0	1	ns
**BCOR**			
SV-Protein-Change (mutation)	0	1	ns
**EPHA3**			
SV-Protein-Change (mutation)	0	1	ns
CNA-COPY-Number (amplification)	0	2	ns
**PRKAR1A**			
CNA-COPY-Number (amplification)	0	2	ns
**RAD51C**			
CNA-COPY-Number (amplification)	0	1	ns
**SPOP**			
CNA-COPY-Number (amplification)	0	2	ns
**RB1**			
CNA-Ratio (loss)	1	0	ns
**DOT1L**			
SV-Protein-Change (mutation)	1	0	ns

CNA-COPY-Number (amplification)	1	0	ns
**NBN**			
CNA-COPY-Number (amplification)	1	1	ns
**ATM**			
SV-Protein-Change (mutation)	1	2	ns
**DIS3**			
SV-Protein-Change (mutation)	1	0	ns
**NRAS**			
CNA-COPY-Number (amplification)	0	1	ns
**TERC**			
CNA-COPY-Number (amplification)	0	1	ns
**CSF1R**			
SV-Protein-Change (mutation)	0	1	ns
**INPP4B**			
SV-Protein-Change (mutation)	0	1	ns
**WHSC1**			
SV-Protein-Change (mutation)	0	1	ns
**HSD3B1**			
CNA-COPY-Number (amplification)	0	2	ns

**MAP2K4**			
SV-Protein-Change (mutation)	0	1	ns
MKNK1			
SV-Protein-Change (mutation)	0	1	ns
**MSH6**			
SV-Protein-Change (mutation)	0	2	ns
**TSC2**			
SV-Protein-Change (mutation)	1	1	ns
**MUTYH**			
SV-Protein-Change (mutation)	0	1	ns
**FANCL**			
SV-Protein-Change (mutation)	0	1	ns
**GNA11**			
SV-Protein-Change (mutation)	0	1	ns
**NTRK2**			
SV-Protein-Change (mutation)	0	1	ns
**PIK3C2G**			
SV-Protein-Change (mutation)	0	1	ns
**PIK3R1**			

CNA-COPY-Number (amplification)	0	2	ns
**WHSC1L1**			
CNA-COPY-Number (amplification)	0	2	ns
**ZNF703**			
SV-Protein-Change (mutation)	1	0	ns
CNA-COPY-Number (amplification)	0	1	ns
**BRD4**			
SV-Protein-Change (mutation)	0	1	ns
**CIC**			
SV-Protein-Change (mutation)	0	1	ns
**MED12**			
SV-Protein-Change (mutation)	0	4	ns
**SMAD4**			
SV-Protein-Change (mutation)	1	0	ns
**NOTCH3**			
SV-Protein-Change (mutation)	1	0	ns
**FAM123B**			
SV-Protein-Change (mutation)	1	0	ns
**FANCC**			
SV-Protein-Change (mutation)	1	0	ns
**GNA13**			
SV-Protein-Change (mutation)	1	0	ns
**HGF**			
SV-Protein-Change (mutation)	1	0	ns
**PALB2**			
SV-Protein-Change (mutation)	1	0	ns
**PDCD1**			
SV-Protein-Change (mutation)	1	0	ns
**POLE**			
SV-Protein-Change (mutation)	2	0	ns
**ROS1**			
SV-Protein-Change (mutation)	2	1	ns
**FOXL2**			
SV-Protein-Change (mutation)	1	0	ns
**STK11**			
SV-Protein-Change (mutation)	1	0	ns
**MST1R**			
SV-Protein-Change (mutation)	1	0	ns
CNA-COPY-Number (loss)	0	1	ns
**NOTCH2**			
CNA-COPY-Number (amplification)	1	1	ns
**RARA**			
CNA-COPY-Number (amplification)	0	3	ns
**ARID1A**			
SV-Protein-Change (mutation)	2	2	ns
**CUL3**			
SV-Protein-Change (mutation)	1	0	ns
**FH**			
SV-Protein-Change (mutation)	1	0	ns
**FBXW7**			
SV-Protein-Change (mutation)	1	0	ns
**CHEK2**			
SV-Protein-Change (mutation)	1	0	ns
**TYRO3**			
SV-Protein-Change (mutation)	1	0	ns
SV-Protein-Change (mutation)	0	1	ns
**CDK4**			
SV-Protein-Change (mutation)	0	1	ns
**ESR1**			
SV-Protein-Change (mutation)	1	1	ns
**KEL**			
SV-Protein-Change (mutation)	0	1	ns
**TGFBR2**			
SV-Protein-Change (mutation)	0	1	ns
**ERBB3**			
SV-Protein-Change (mutation)	0	1	ns
**JAK3**			
SV-Protein-Change (mutation)	0	1	ns
**CASP8**			
SV-Protein-Change (mutation)	1	0	ns
**ERBB4**			
SV-Protein-Change (mutation)	1	0	ns
